# A Highly Efficient Gene Expression Programming (GEP) Model for Auxiliary Diagnosis of Small Cell Lung Cancer

**DOI:** 10.1371/journal.pone.0125517

**Published:** 2015-05-21

**Authors:** Zhuang Yu, Haijiao Lu, Hongzong Si, Shihai Liu, Xianchao Li, Caihong Gao, Lianhua Cui, Chuan Li, Xue Yang, Xiaojun Yao

**Affiliations:** 1 The Affiliated Hospital of Qingdao University, Department of Oncology, Qingdao, Shandong, P.R. China; 2 Institute for Computational Science and Engineering, Laboratory of New Fibrous Materials and Modern Textile, the Growing Base for State Key Laboratory, Department of Pharmacy, Qingdao University, Qingdao, Shandong, P.R. China; 3 The Affiliated Hospital of Qingdao University, The Central Laboratory, Qingdao, Shandong, P.R. China; 4 Department of Pharmacy, Qingdao University, Qingdao, Shandong, P.R. China; 5 Department of Public Health, Qingdao University Medical College, Qingdao, Shandong, P.R. China; 6 The Affiliated Hospital of Qingdao University, Department of Thoracic Surgery, Qingdao, Shandong, P.R. China; 7 Department of Chemistry, Lanzhou University, Lanzhou, Gansu, P.R. China; University Medical Center of Princeton/Rutgers Robert Wood Johnson Medical School, UNITED STATES

## Abstract

**Background:**

Lung cancer is an important and common cancer that constitutes a major public health problem, but early detection of small cell lung cancer can significantly improve the survival rate of cancer patients. A number of serum biomarkers have been used in the diagnosis of lung cancers; however, they exhibit low sensitivity and specificity.

**Methods:**

We used biochemical methods to measure blood levels of lactate dehydrogenase (LDH), C-reactive protein (CRP), Na^+^, Cl^-^, carcino-embryonic antigen (CEA), and neuron specific enolase (NSE) in 145 small cell lung cancer (SCLC) patients and 155 non-small cell lung cancer and 155 normal controls. A gene expression programming (GEP) model and Receiver Operating Characteristic (ROC) curves incorporating these biomarkers was developed for the auxiliary diagnosis of SCLC.

**Results:**

After appropriate modification of the parameters, the GEP model was initially set up based on a training set of 115 SCLC patients and 125 normal controls for GEP model generation. Then the GEP was applied to the remaining 60 subjects (the test set) for model validation. GEP successfully discriminated 281 out of 300 cases, showing a correct classification rate for lung cancer patients of 93.75% (225/240) and 93.33% (56/60) for the training and test sets, respectively. Another GEP model incorporating four biomarkers, including CEA, NSE, LDH, and CRP, exhibited slightly lower detection sensitivity than the GEP model, including six biomarkers. We repeat the models on artificial neural network (ANN), and our results showed that the accuracy of GEP models were higher than that in ANN. GEP model incorporating six serum biomarkers performed by NSCLC patients and normal controls showed low accuracy than SCLC patients and was enough to prove that the GEP model is suitable for the SCLC patients.

**Conclusion:**

We have developed a GEP model with high sensitivity and specificity for the auxiliary diagnosis of SCLC. This GEP model has the potential for the wide use for detection of SCLC in less developed regions.

## Introduction

Lung cancer is a major cause of cancer death worldwide, representing about 12.7% (1.6 million cases) of all new cancer cases each year and 18.2% (1.4 million deaths) of all cancer deaths[1]. It has a poor prognosis, with a 15% 5-year survival rate, and more than 75% of patients are diagnosed at late stages of the disease[2,3]. Small cell lung cancer (SCLC) is one of the major types of lung cancer, with the highest degree of malignancy. Current therapy methods, such as chemotherapy, radiotherapy, and surgery are very limited for the treatment of late stage SCLC. Although tremendous effort and progress have been made in the treatment of lung cancer, recent advances in early detection have led to small improvements in prognosis[4]. Therefore, an effective screening method for the early diagnosis of SCLC is critically important for increasing clinical diagnosis effectiveness and outcome of this disease.

Many different techniques have been used in the detection of lung cancers, including Chest Radiograph (x-ray), Computed Tomography (CT), Magnetic Resonance Imaging (MRI), Sputum Cytology, and bronchoscopy[5]. In recent years, whole-body positron-emission tomography (PET) has emerged to simplify and improve the evaluation of patients with this type of tumor[6]. However, these techniques are invasive, expensive, and/or time-consuming. For example, bronchoscopy can cause damage to the bronchus and lung. In addition, these detection methods are not sufficiently sensitive and specific enough in most cases[7,8] and misdiagnosis of indolent tumors, due to the low specificity of these methods, may lead to unnecessary surgical treatments[9,10]. In order to avoid overtreatment of the disease, non-invasive blood tests have been widely used in clinical settings for screening of SCLC. Biomarkers are molecules in blood, other body fluids, or tissues that can be used to evaluate the normal and abnormal conditions of human beings. Biomarkers can complement or replace radiological examinations for the screening of cancers or routine clinical visits[11,12]. In lung cancer, biomarker evaluations have been conducted in serum, tissue, and sputum [12]. Several serum biomarkers, including the carcinoembryonic antigen (CEA), the cytokeratin 19 fragment (CYFRA 21–1), the tissue polypeptide antigen (TPA), the squamous cell carcinoma antigen (SCC), the cancer antigen 125 (CA-125), the cancer antigen 153 (CA-153), the pro-gastrin-releasing peptide (ProGRP), the cancer antigen 199 (CA-199), tumor-associated glycoprotein 72–3 (TAG-72.3) and neuron-specific-enolase (NSE), have shown usefulness for diagnosis of lung cancers[13][14][15]. Nevertheless, each of them has failed to demonstrate the requisite sensitivity and specificity as a diagnostic tool to warrant clinical development[8]. The combination of a number of biomarkers may improve the diagnostic efficiency of cancers[16]. However, the combined use of tumor biomarkers is not widely used, especially in small hospitals and in less developed countries, because of the high cost of equipment and reagents. In this study, we have found the combination of economical efficiency and correlative serum such as LDH, CRP, Na^+^, Cl^-^, which can be obtained by common biochemical detection method and don’t need exorbitant agentias or facilities. In a rural and impoverished area, using the approach, a fundamental serum test could warn people who are at higher risk of suffering from cancer and to do an indepth health examination such as CT, PET-CT and so on.

Therefore, new technology is urgently needed to find the association information between a large set of biomarkers and for the early detection of lung cancer. In recent years, with the development of science and technology, computer-aided design has become an auxiliary tool for the diagnosis of human cancers. Nowadays, machine learning methods, such as artificial neural networks (ANNs), decision trees, the naive bayesian (NB) algorithm, and support vector machines (SVM) have been utilized in the diagnosis and prognosis prediction of cancers[17]. For instance, ANNs of different EGFR microdeletion mutations have been used to improve the diagnosis efficiency of non-small cell lung cancer (NSCLC)[18]. The ANN model combined with six tumor biomarkers, including CEA, gastrin, NSE, sialic acid (SA), Cu/Zn, and Ca, was used to successfully differentiate lung cancer from benign lung disease, a normal control, and gastrointestinal cancers[19]. A previous study has shown that NB techniques are useful for diagnosis and to generate treatment recommendations and predict the 1-year-survival rate in lung cancer patients[20]. The combination of protein characteristics and attribute weighting models with a support vector machine (SVM) was used to discriminate SCLC and NSCLC[21]. These methods have led to the development of classifiers that are capable to discriminate between cancer and non-cancer samples. The ANNs, SVMs and NBs have been widely used for classification problems[17][20][22]. The ANNs have the ability to fulfill the statistical that contain linear, logistic and nonlinear regression, but it is hard for ANNs to understand the structure of algorithm，due to that ANNs are a “black-box” technology and hence, they can hardly discover how to operate the classification. Otherwise, generous attributes cause overfitting easily [17]. Contrast to ANNs, in SVM the overfitting hardly occur, but the training is slow when inputing large number of data. The NB is very easy to discern but like ANN excessive attributes can misinform the classification[17][23]. Recently, a novel evolutionary algorithm called Gene Expression Programming (GEP) which is an automatic programming approach first introduced by Ferreira[24] was studied for auxiliary diagnosis of cancers. GEP has the advantages of flexibility and the power to explore the entire search space, which comes from the separation of genotype and phenotype and has the visualization data model. It is easy to implement and point out why GEP can not work via parameter adjustment [24][25][26]. One particular study has manifested the superior value of GEP in predicting the adverse events of radical hysterectomy in cervical cancer patients with an accuracy of 71.96% [27]. In our fundamental research, classification of lung tumors was made based on biomarkers (measured in 120 NSCLC and 60 SCLC patients) by setting up optimal biomarker joint models with GEP algorithm [28]. However, there is little relevant data regarding GEP applied to lung cancer so far.

In this study, we developed a prediction model using the GEP method to improve the diagnostic efficacy of SCLC. A number of biomarkers have previously been demonstrated to be useful for lung cancer diagnosis. Our GEP model suggested a novel multi-analysis of serum biomarkers for the early detection of SCLC.

## Materials and Methods

### Patients and controls

**Fig 1:**
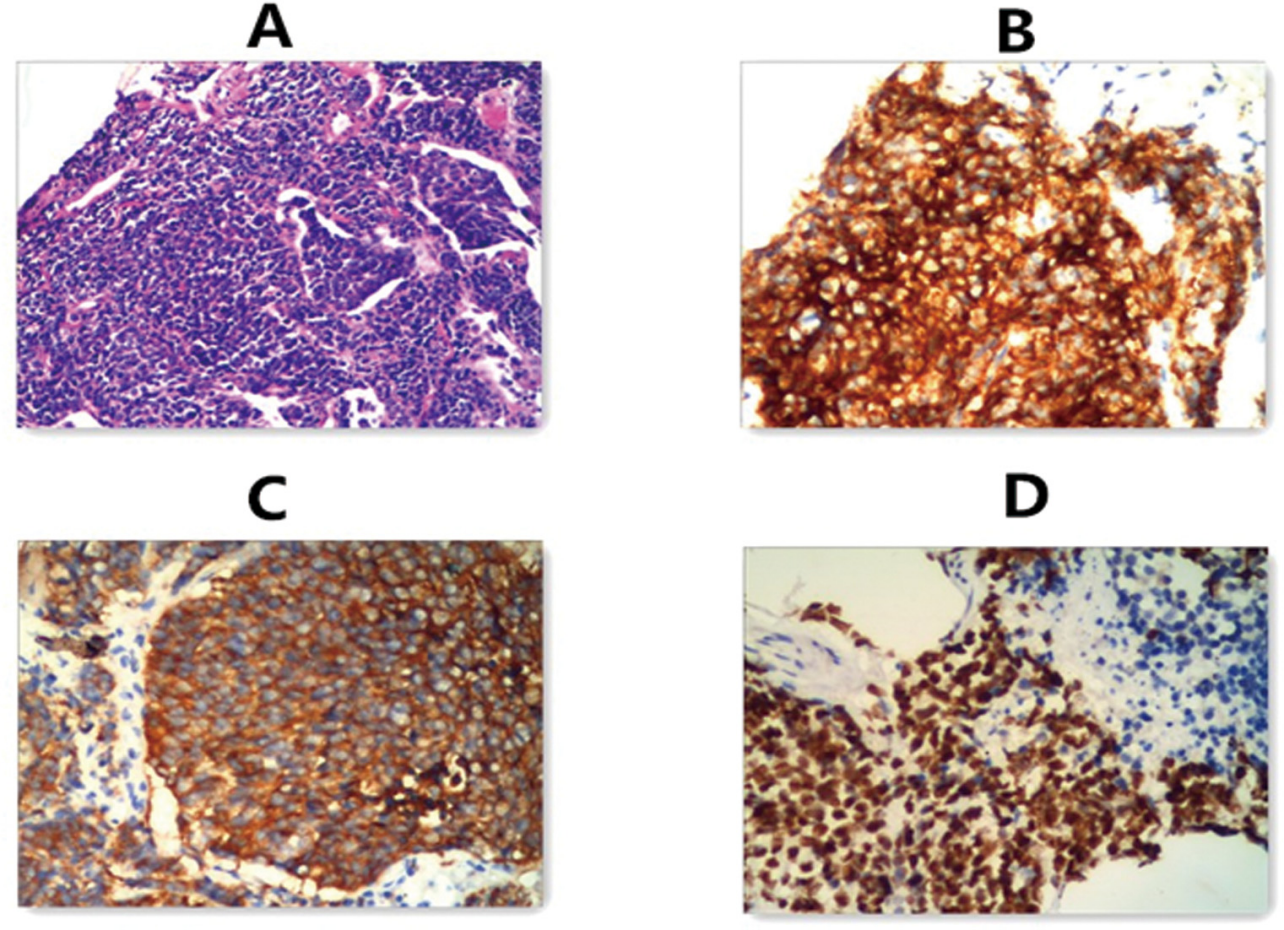
Histopathologic test of SCLC patients. A. hematoxylin-eosin staining of biopsy specimen slice. B. CD56(+) findings in immunohistochemical method. C. Syn (+) findings in immunohistochemical method. D.TTF-1(+) findings in immunohistochemical method

In total 430 cases, including 145 SCLC patients, 130 non-small cell lung cancer (NSCLC) patients and 155 non-cancer controls, were enrolled from the Affiliated Hospital of Qingdao University between July of 2006 and May of 2013. The diagnosis of 145 SCLC patients was based on biopsy and histopathology, and they were proven to be untreated primary lung cancers (Fig 1), the 130 NSCLC patients were diagnosed with primary tumor in stage I, II before surgery. Histological diagnosis of primary lung cancer was established according to the revised classification of lung tumors by the World Health Organization and the International Association for Lung Cancer Study[29].

The SCLC group included 94 male and 51 female patients, aged between 33 and 78 years old. The control group was composed of 155 non-cancer cases, which underwent examinations proving their health (86 males and 69 females). The NSCLC patients (69 males and 61 females) were included in the negative control to show the difference from SCLC, we selected 130 cases from 155 non-cancer cases as the healthy control. Research approval was obtained from the corresponding ethics committee and written informed consent was obtained from all participants. Samples and health information were labeled using unique identifiers to protect subject confidentiality (Tables 1 and 2).

**Table 1:** Demographic and clinical profiles of SCLC patients and controls included in this study ($\bar{x}\pm s$).

Demographic profile**	Controls (n = 155)	SCLC (n = 145)	p-value*
Age (years)	56.23±8.72	57.92±9.46	*0*.*270*
Range (age)	29–81	33–78	-
Sex (F/M)	69/86	51/94	*0*.*099*
SCLC	-	145	-
Stage (L/E)	-	74/71	-
Smoking	86/69	92/53	*0*.*161*

*Statistics were conducted using the independent-Samples T Test and chi-square test.

**F = female and M = for male. L = limited stage and E = extensive stage.

**Table 2:** Demographic and clinical profiles of NSCLC patients and controls included in this study ($\bar{x}\pm s$).

Demographic profile	Controls (n = 130)	NSCLC (n = 130)		p-value*
Age (years)	56.24±8.94	57.75±10.69	*0*.*17*	
Range (age)	29–81	21–80	-	
Sex				
Male	64	69	*0*.*385*	
Female	66	61		
Stage (I,II)	-	130	-	
Smoking	64/66	72/58	*0*.*987*	

*Statistics were conducted using the independent-Samples T Test and chi-square test.

### Selection of six serum biomarkers

We selected six biomarkers that are closely related to lung cancers, especially to SCLC, and that have been widely used in the screening of SCLC. The indexes we chose have been incorporated into the GEP model. Based on previous clinical examination, the serum levels of LDH and CRP in SCLC patients are significantly higher than in healthy controls, but the serum level of sodium and chloride are significantly lower than that in normal controls. The serum level of LDH, which is commonly elevated in neoplastic disorders, has been suggested as a powerful tumor marker for many years. Therefore, these markers have significant meaning in SCLC. For example, lung cancer patients, especially SCLC patients, the Syndrome of Inappropriate Anti Diuretic Hormone secretion (SIADH) is considered to be the leading cause for hyponatraemia and hypochloraemia and can be induced by comorbidity such as lung cancer. Also, the major osmotic active substances that in the extracellular fluid principal contain serum sodium and its accompanying anions chloride[30][31]. There are also numerous reports on the association between chronic inflammation and cancer[32]. CRP is a nonspecific acute-phase inflammatory response serum marker produced by hepatocytes under the regulation of interleukin (IL)-6[33]. CEA and NSE are the most common biomarkers used in lung cancer screening in hospital[34][35].

### Measurements of serum biomarkers

Blood (10 ml) was collected in serum separator tubes, processed immediately, and separated by centrifugation at 3,000 rpm at room temperature for 10 minutes. The separated serum was then aliquoted and stored at -80°C for the measurement of the six biomarkers mentioned above. CEA and NSE were determined by electro-chemiluminescence immunoassay (ECLIA), using the Roche E601 chemical luminescence immunity analyzer with the auxiliary reagent kit (Dongying J&M Chemical Co., Ltd., China). LDH, CRP, Na^+^, and Cl^-^ were measured by polyacrylamide gel electrophoresis (PAGE), immunoturbidimetry (ITM), and ion selective electrode methods, respectively, using the Hitachi 7600–020 automatic biochemical analyzer (Beijing Leadman Biochemical Technology Company, Beijing, China). Results were presented as mean values of duplicates after the subtraction of background values. The normal critical values of LDH (99–245 u/l), CRP (0-3mg/l), Na^+^ (136–146 mmol/l, Cl^-^ (96-108mmol/l), CEA (0–3.4 ng/ml), and NSE (0-17ng/ml) were used as standards.

### Gene expression programming (GEP) models

**Fig 2:**
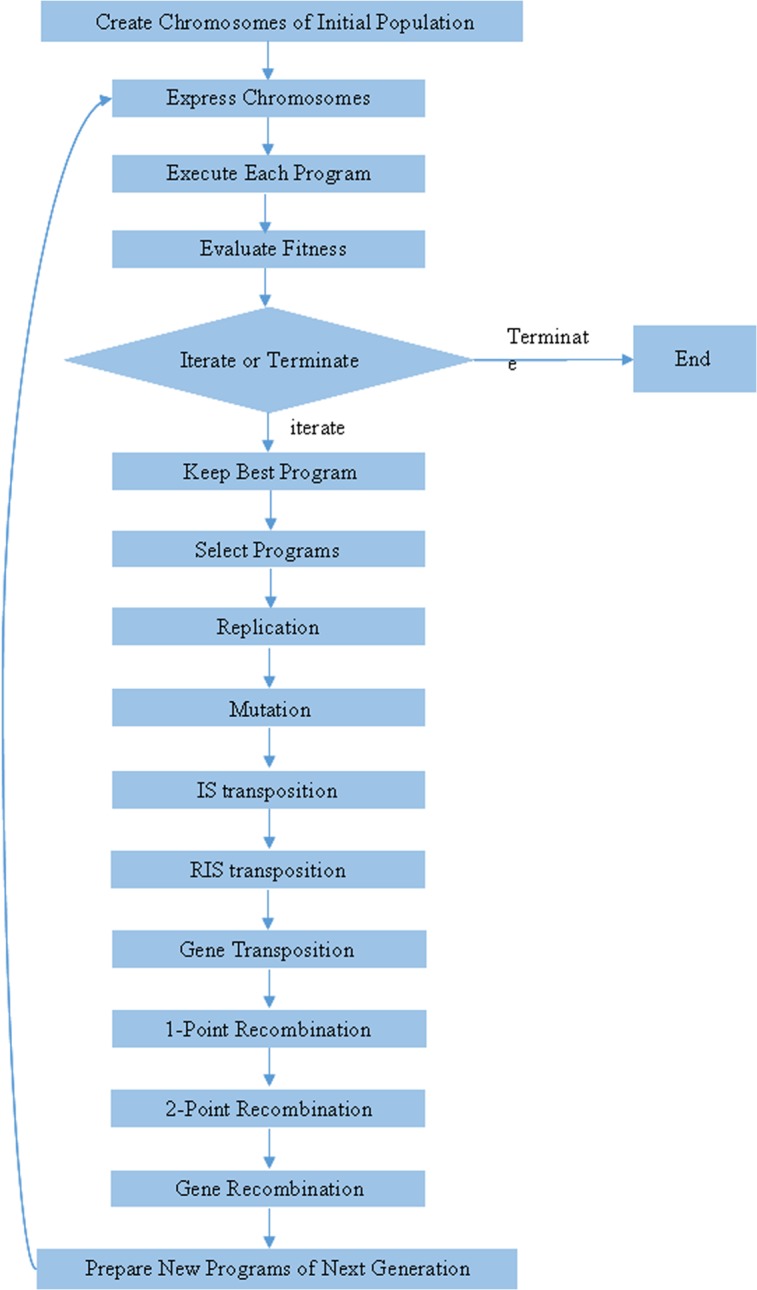
The flowchart of the GEP modeling in this study.

GEP is an evolutionary algorithm introduced by Ferreira in 2001[25]. It can emulate biological evolution based on computer programming. With the assumption of being, in some way, a natural development of genetic programming (GP) preserves few properties of genetic algorithms (GA)[36][37]. The GEP algorithm inherits the advantages of GA and GP, but overcomes their disadvantages. In contrast to GP, the chromosomes in GEP are not represented as trees, but as linear strings of fixed length, with features taken from GA. GEP adopts a simple linear fixed-length manner to describe individuals; it is therefore easy to use a nonlinear tree structure to solve complicated nonlinear problems, thus achieving the purpose of using simple coding to solve complex problems[38]. GEP uses characteristic linear chromosomes, which are composed of the genes structurally organized in the head and the tail. Head may contain functional elements like {Q, +, −, ×, /} or terminal elements like, “Q” is the statistical function of square root. The size of the tail (t) is computed as t = h (n-1) + 1, where n is the maximum number of parameters required in the function set[39]. When the representation of each gene is given, the genotype is established. It is then converted to the phenotype expression tree (ET). The chromosomes function is used as a genome and is modified by means of mutation, transposition, root transposition, gene transposition, gene recombination, and one- and two-point recombination. The flowchart of a gene expression algorithm (GEA) is shown in Fig 2. [24].

The algorithm begins with the random creation of the chromosomes in the initial population. Then the chromosomes are expressed and the fitness of each individual is evaluated. According to fitness, reproduction with modification is made, the individuals are then selected and the results lead to new traits. Additionally, the individuals of this new generation are subjected to the same developmental process: expression of the genomes, confrontation of the selection environment, and reproduction with modification. It is repeated for a certain number of generations until a satisfying solution has been found. It is important that the individuals are selected and copied into the next generation according to the fitness by roulette wheel sampling with elitism. This guarantees the survival and cloning of the best individual to the next generation. Each GEP gene contains a list of symbols with a fixed length that can be any element from a function set [36]:

{+;−;*;/;≤;≥;>;=;<;sqr;sqrt;exp;ln;cos;sin;tan} (1)

The optimum fitness is:

$$\begin{array}{c} fitness(i)=\frac{TP+TN}{TP+FN+TN+FP} \end{array}$$ (2)

$$Sensitivity=\frac{TP}{TP+FN}$$ (3)

$$Specificity=\frac{TN}{TN+FP}$$ (4)

TP, TN, FP, FN are the number of true positives (TPs), true negatives (TNs), false positives (FPs), and false negatives (FNs), respectively.

### The theory of ANN models

Artificial Neural Networks (ANNs) that has the ability of classification is a mathematical model, which original designed to imitate human neural system. Multiple neurons interconnect to each other and arranged in to a wiring layer. ANNs use complicated layers (called hidden layers) to deal input and output, the input where each neuron represents an independent variable. ANNs contain a series of different architectures including Multilayer Perceptron (MLP) and Radial Basis Function (RBF) [17][39]. MLP employs the back-propagation learning algorithm and a non-linear function to transmit the sum. RBF network activates neuron in hidden layer through radial basis function which has two parameters: the center location of the function and its bias. In RBF network, the hidden layer accepts input data via an unsupervised form[40].

### Statistical analyses

Statistical analyses were performed using SPSS 16.0. Differences between groups were calculated by means of a nonparametric Wilcoxon test (Mann–Whitney U test), independent-Samples T Test and chi-square test. *P values < 0*.*05* were considered to be statistically significant.

### Detection capability comparison

The Receiver Operating Characteristic (ROC) curves were used to describe sensitivity of biomarkers, alone and combined, which were graphed by “R programming project 2.15–1”. Using ANNs to compare the detection capability, we can ascertain the optimal algorithm.

### Ethics statement

Research approval was obtained from the Ethics Committee of Qingdao University Medical College and written informed consent was obtained from all participants. The study were followed by the STARD (Standards for Reporting of Diagnostic Accuracy) checklist to improve the accuracy and completeness of reporting of studies of diagnostic accuracy[41].

## Results

### Demographic and clinical profiles, as well as the serum levels of six biomarkers of SCLC patients and normal controls

The clinical characteristics of SCLC patients and normal controls were summarized in Table 1, the NSCLC patients and controls were in Table 2. No significant differences of age and smoking history were observed between these two groups. To establish a novel multiple-analysis of serum biomarkers for efficient screening of SCLC, a set of six biomarkers were selected and their serum concentrations were determined by 145 lung cancer patients and 155 control subjects (S1 Dataset). SCLC patients exhibited significantly higher concentrations of serum LDH, CRP, CEA, and NSE than normal controls (*p <0*.*001*), whereas the concentrations of Na^+^ and Cl^-^ were significantly lower than in normal controls (*p <0*.*001*) (Table 3). There are significant differences in the concentrations of LDH, Na, Cl and NSE between SCLC and NSCLC means that these biomarkers are particularly suitable for SCLC (Table 4). The correlation analysis depended on Spearman rank correlation analysis was to exclude potential confounders, the correlation coefficient which is close to “1” means repetitive in the GEP models, the six biomarkers perform their mission well and have significant role respectively. (Table 5).

**Table 3:** Serum levels of six biomarkers in SCLC patients and control subjects.

biomarker	Controls (n = 155)		SCLC (n = 145)		Z-value	P-value*
	Median	Range	Median	Range		
LDH(u/l)	146	55–397	180	3–801	-6.506	*<0*.*0001*
CRP(mg/l)	1.36	0.04–18.2	6.18	0.04–117.96	-8.57	*<0*.*0001*
Na^+^ (mmol/l)	142.47	127–146.83	140	101.4–146.1	-6.614	*<0*.*0001*
Cl^-^ (mmol/l)	105	98–111	102	78–137.8	-7.328	*<0*.*0001*
CEA(ng/ml)	2.07	0.2–14.66	4.29	0.08–181	-7.421	*<0*.*0001*
NSE(ng/ml)	12.44	6.76–38.19	24.27	1.07–370	-5.081	*<0*.*0001*

* Statistics were conducted using the non-parametric Wilcoxon test (Mann–Whitney U test).

**Table 4:** Serum levels of six biomarkers in SCLC patients and NSCLC patients.

biomarker	NSCLC(n = 130)		SCLC (n = 145)		Z-value	P-value*
	Median	Range	Median	Range		
LDH(u/l)	159.98	10–540	180	3–801	-5.043	*<0*.*0001*
CRP(mg/l)	19.55	0–145	6.18	0.04–117.96	-0.515	*0*.*607*
Na^+^ (mmol/l)	141.57	134–146	140	101.4–146.1	-4.777	*<0*.*0001*
Cl^-^ (mmol/l)	102.60	1–110	102	78–137.8	-4.351	*<0*.*0001*
CEA(ng/ml)	52.66	0–781	4.29	0.08–181	-2.857	*0*.*010*
NSE(ng/ml)	13.34	1–40	24.27	1.07–370	-4.728	*<0*.*0001*

* Statistics were conducted using the non-parametric Wilcoxon test (Mann–Whitney U test).

**Table 5:** The correlation analysis of the biomarkers were depended on Spearman rank correlation analysis (r = correlation coefficient, P value *of 0 <0*.*0001*).

	LDH	CRP	Na	Cl	CEA	NSE
LDH	1	0.302	0.006	0.049	0.289	0.295
CRP		1	0.161	0.199	0.093	0.063
Na			1	0.705	0.025	0.054
Cl				1	0.038	0
CEA					1	0.109
NSE						1

### ROC curves analyses to represent sensitivity/specificity of each biomarker and their combinations

**Fig 3:**
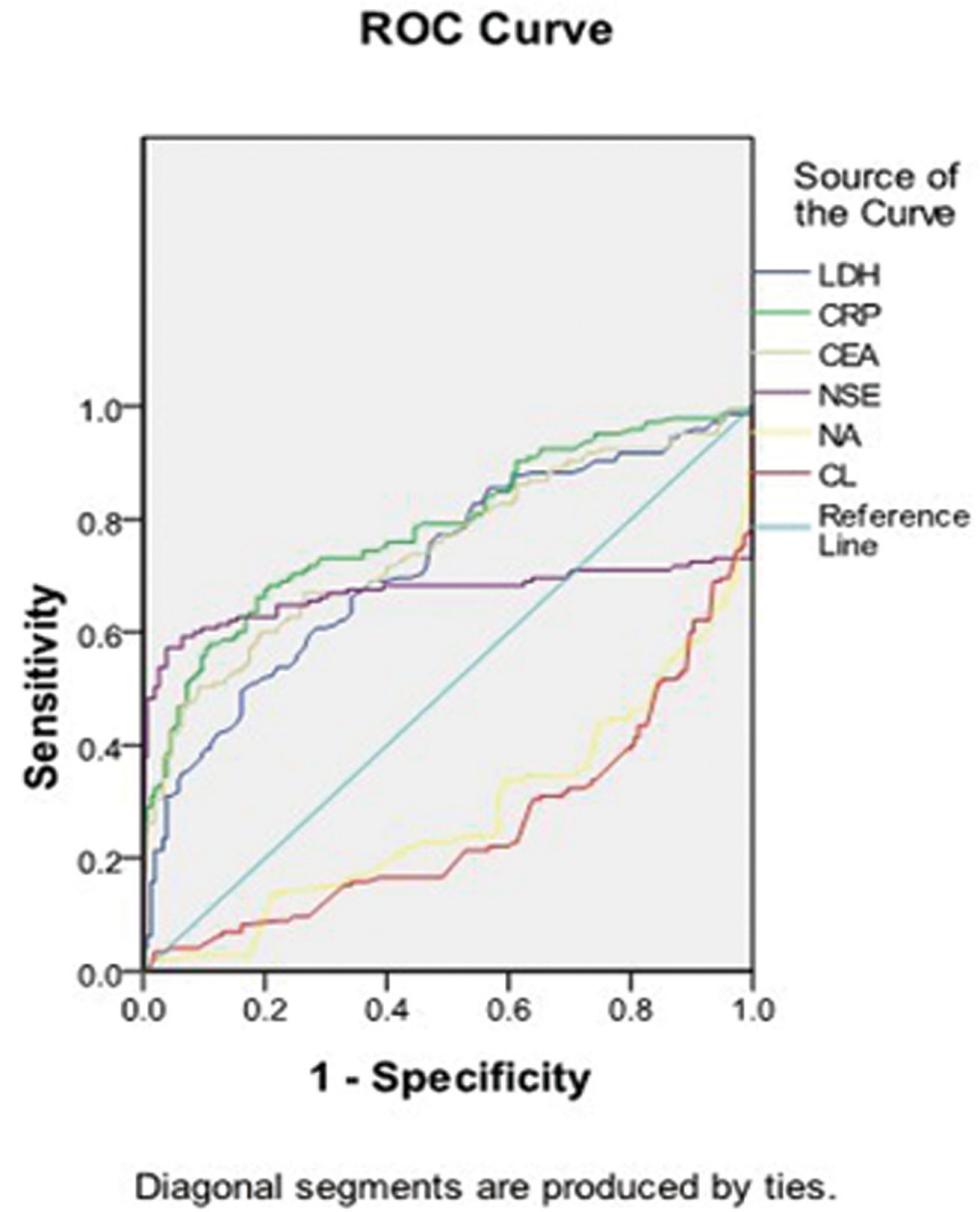
ROC curves analyses to represent sensitivity/specificity of each biomarker, and the Area Under the Curve represents: LDH = 0.717, CRP = 0.786, CEA = 0.748, NSE = 0.670, Na^+^ = 0.279, Cl^-^ = 0.255.

**Fig 4:**
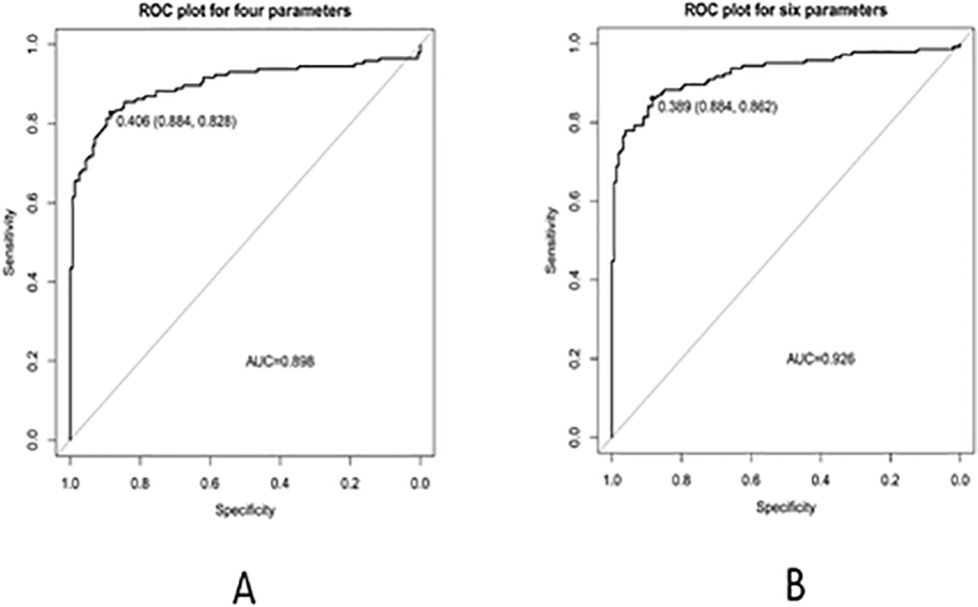
Comparison of the performance (sensitivity) from combined biomarkers, A is trained with six biomarkers and B is trained with four biomarkers. The sensitivity trained by six biomarkers combination performed better than four biomarkers.

The ROC curves to discover the sensitivity/specificity in each biomarker were determined by comparison with the area under the curve, we found the result in serum sodium and serum chloride were lower than any other biomarkers (Fig 3), then build models dividing two groups to confirm whether Na^+^ and Cl^-^ are meaningful in the detection of lung cancer patients and controls. Model 1 has united all the six biomarkers and model 2 has conjoined four biomarkers that remove serum sodium and serum chloride. The striking difference of the performance in model 1 and model 2 was graphed in Fig 4, the model 1 with 6 biomarkers in the ROC curve has a significant advantage (Fig 4).

### GEP modeling

#### GEP model 1 incorporating six serum biomarkers

A software known as “Automatic Problem Solver 3.0” was used to run the algorithm. The GEP modeling randomly selected four of five partitions as a training set (240 subjects) for model generation, including 115 SCLC patients and 125 normal controls. Next, the GEP parameters were modified to test the remaining 60 subjects for model validation. The concentration of six biomarkers was input into the GEP model to calculate its detection sensitivity and specificity for the discrimination of SCLC and normal controls. GEP model 1 used all six biomarkers as inputs and the algorithm was:

$$\begin{array}{l} \text{y}=\frac{x_{0}-x_{4}}{x_{5}\times \sqrt{\log_{10}\;x_{0}}}+\frac{e^{apsLogi(x_{3})}+x_{4}}{apsLogi(x_{5})}+x_{2}\times apsLogi(apsLogi(\frac{1}{apsLogix_{0}}+x_{4}))\\ +e^{apsLogi(\log_{10}\;x_{3})}+x_{1}-x_{2}+x_{5} \end{array}.$$

If the calculated value of “y” equal to or greater than the rounding threshold, then the record is classified as "1", "0" otherwise. The variables x_0_, x_1_, x_2_, x_3_, x_4_, and x_5_ represented the biomarkers LDH, CRP, Na^+^, Cl^-^, CEA, and NSE, respectively.

Patients suffered from lung cancer were marked as class “1”, while the healthy subjects were marked as class “0”. The serum concentrations of LDH, CRP, Na^+^, Cl^-^, CEA, and NSE were used as inputs in model 1. The general experiment setup was summarized in Table 6. This model successfully discriminated 281 out of 300 subjects, which represented a determination coefficient of 93.75% (225/240) and 93.33% (56/60) for training and test sets, respectively (S1 Dataset).

**Table 6:** SCLC detection rate of GEP model 1 and model 2.

	Model 1		Model 2	
	Training set	Test set	Training set	Test set
	n = 240	n = 60	n = 240	n = 60
Accuracy	93.75%	93.33%	93.75%	91.67%
Sensitivity	92.17%	93.33%	89.57%	86.67%
Specificity	95.20%	93.33%	97.60%	96.67%
Error	6.25%	6.67%	6.25%	8.33%
CC	0.87	0.87	0.88	0.84
MSE	0.06	0.07	0.06	0.08
RAE	0.13	0.12	0.13	0.17
MAE	0.06	0.06	0.06	0.08
RSE	0.25	0.27	0.25	0.33

CC = Correlation Coefficient; MSE = Mean Squared Error; RAE = Root Mean Squared Error; MAE = Mean Absolute Error; RSE = Relative Squared Error.

#### GEP model 2 including four biomarkers

While the performance of model 1 with 6 biomarkers was good, we wanted to ascertain whether the numbers of biomarkers could be decreased to only four, which could significantly reduce the cost and time for SCLC screening. In model 2, we only chose the markers that were widely used in the detection of lung cancer, including LDH, CRP, CEA, and NSE, with the same function set described above.

The algorithm of GEP model 2 was:

$$\begin{array}{l} y=\sqrt{e^{x_{3}}\times x_{3}\times x_{2}}+\sqrt{apsLogi(x_{0})}+x_{0}\times x_{3}\times (x_{1}+x_{2}-1)-\log_{10}\;x_{3}\\ +x_{0}^{2}\times (\log_{10}\;x_{0}+x_{2}-x_{3}+e^{\frac{x_{1}}{x_{3}}}) \end{array}$$

$$apsLogi(x)=\frac{1}{1+e^{-x}}$$

If the calculated value of “y” equal to or greater than the rounding threshold, then the record is classified as "1", "0" otherwise. In this model, variables x_0_, x_1_, x_2_, and x_3_ were biomarkers LDH, CRP, CEA, and NSE, respectively.

The accuracy of GEP model 2 was 91.66% and the sensitivity was 86.67% in the test set, which was lower than that in model 1 (Table 7). All trainings were made in triplicate to assure that the best architecture was chosen. We have made other combinations to make sure the model 1 is the optimized biomarker panel that acquired the supreme predicted value.

**Table 7:** Parameter settings for the GEP algorithm.

Parameter	Settings
General	
Chromosomes	100
Genes	5
Head size	8
Gene size	17
Linking function	Addition
Function set	+ － * / Exp Sqrt Log Logi Inv
Complexity increase	
Generations without change	200
Number of tries	3
Max. complexity	5
Genetic operators	
Mutation rate	0.044
Inversion rate	0.1
IS transposition rate	0.1
RIS transposition rate	0.1
One-point recombination rate	0.3
Two-point recombination rate	0.3
Gene recombination rate	0.1
Gene transposition rate	0.1
Numerical constants	
Constants per gene	10
Data type	Floating-point
Lower bound	-10
Upper bound	10

### Development of model by Artificial Neural Networks

**Fig 5:**
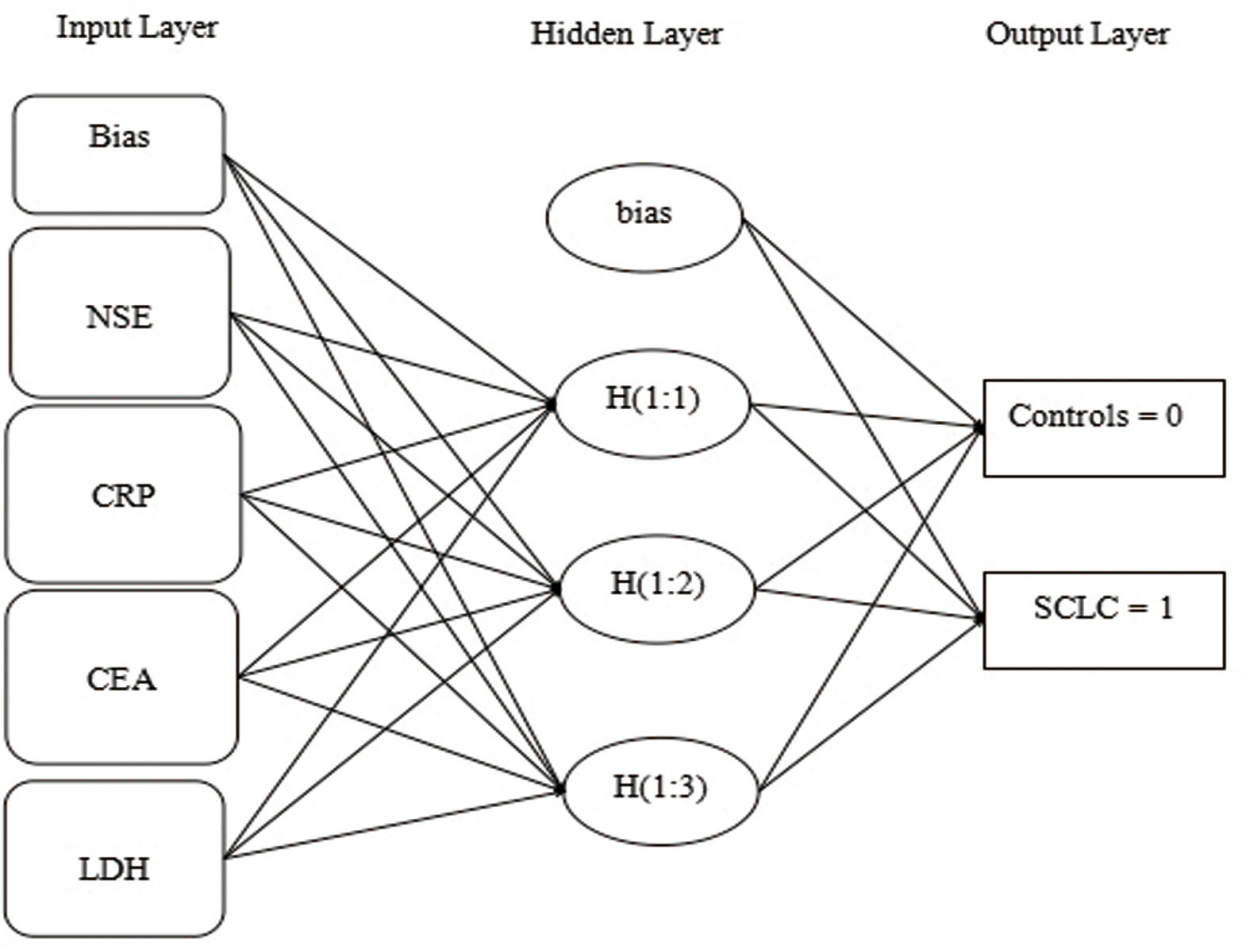
The structure of the ANNs implemented.

In order to compare the classification power between GEP and ANN, IBM SPSS Statistics 18.0 was applied to build ANNs (MLP and RBF models) prediction models. The model1 and model2 were as same to GEP. SCLC patients and controls (0 or 1) were input as a dependent variable as GEP models. Using model 1, MLP indicated accuracy of 85.4%, 80.0% and in RBF acquired an accuracy of 80.0%, 78.3% for training and test phase, respectively. In addition, in model 2 the correct classification rate for MLP represented the identification of 83.3% and 83.3% and for RBF was for 84.2%, 83.3% among training and testing stages, respectively. The software have been ran three times and covariant was different arrange to select the best (Table 8) (Fig 5).

**Table 8:** The detection capability of ANN models in SCLC patients and normal controls.

	Model 1		Model 2	
	Training set	Test set	Training set	Test set
	n = 240	n = 60	n = 240	n = 60
**Accuracy(MLP)**	85.4%	80%	83.3%	83.3%
**Accuracy(RBF)**	80.0%	78.3%	84.2%	83.3%

Compared to ANNs, the GEP algorithm proves the supreme predictive rate which has significant strengths. The ROC curve and GEP model showed that the model 1 is the adequate combination to distinguish lung cancer patients from high-risk people.

#### GEP model 1 incorporating six serum biomarkers performed by limited stage and extensive stage

The optimal GEP model 1 was used to make a comparison between early and late SCLC (74 limited stage and 71 extensive stage). We selected 74 cases from the 155 non-cancer cases as the healthy control. Firstly, in order to explore the early SCLC, as the above method GEP model randomly selected four of five partitions as a training set (118 subjects) for model generation, including 59 early SCLC patients and 59 normal controls. Remaining 30 cases (15 early SCLC and 15 normal controls) were for model validation. It can be observed that the early SCLC acquired the accuracy of 92.37% (109/118) and 90% (27/30) for training and test set, respectively. Secondly, for late SCLC, 116 subjects (57 late SCLC and 59 normal controls) for model generation and 29 cases for model validation, it represented the accuracy of 96.52% (112/116), 91.30% (27/29) for training and test set, respectively. The results showed that the accuracy of late SCLC in GEP model 1 was performed better than early SCLC and total 145 SCLC, but the early SCLC accuracy was close to the result of 145 SCLC, it was still a good performance (S3 Dataset) (S4 Dataset).

#### GEP model 1 performed by NSCLC patients and normal controls

To confirm the GEP model 1 test, NSCLC patients have been included in the negative control with healthy subjects. As the above method, GEP randomly selected 208 subjects (104 NSCLC patients and 104 normal controls) for model generation, 52 subjects (26 NSCLC patients and 26 normal controls) for model validation respectively. It indicated that the accuracy of 87.5% (182/208), 86.5% (45/52) for training and test set, respectively. Meanwhile, the results were significantly worse than SCLC patients and were enough to prove that the GEP model is suitable for the SCLC patients (Table 9) (S2 Dataset).

**Table 9:** The detection capability of GEP model 1 with six biomarkers in SCLC and NSCLC patients.

	SCLC		NSCLC	
	Training set	Test set	Training set	Test set
	n = 240	n = 60	n = 208	n = 52
**Accuracy**	93.75%	93.33%	87.50%	86.53%
**Sensitivity**	92.17%	93.33%	81.73%	84.62%
**Specificity**	95.20%	93.33%	93.26%	88.46%

## Discussion

SCLC accounts for approximately 13–18% of all lung cancers, with diverse incidences in different countries[42]. Without treatment, it has the most aggressive clinical course of all lung cancer types, with survival from 2 to 4 months[43]. Diagnosis of SCLC at its early stage is challenging, because it is usually asymptomatic until advanced stages, which causes poor prognosis[44]. This emphasizes the significance of a reliable early-stage diagnosis method to prolong lives[45].

Various methods have been used for the detection of SCLC, such as thoracic radiography, sputum cytology, and CT. The efficacy of these tools has been evaluated in clinical trials and it turns out that thoracic radiography and sputum cytology have low sensitivity for early-stage detection of SCLC [46,47]. Although CT imaging has emerged as an effective technique for the diagnosis of many human diseases, the most prominent limitation of CT imaging for the detection of lung cancers is the high rate of mistaken benign pulmonary nodules as lung cancers [48,49]. In addition, CT imaging examination is still costly for most people in developing countries and medical insurance agencies would not approve the use of CT scans as a surveillance strategy for lung cancers.

Biological markers can be easily detected in biological fluids using minimally invasive procedures, which can significantly enhance the detection rate of a number of human cancers. Several tumor markers, such as ɑ-fetoprotein (AFP), prostate specific antigen (PSA), and cancer antigen125 (CA125), have been proven to be highly sensitive and effective for the screening of liver, prostate, and ovarian cancers [50]. Each biomarker has low diagnostic because of limited sensitivity and specificity which is partially owing to the heterogeneous of the disease [15,51]. Many tumor markers are not used alone for routine tumor screening because of low detection rates and unacceptable false-positive diagnoses [52]. In this study, some conventional and economical markers such as LDH, CRP, Na^+^, Cl^-^ and other two tumor biomarkers(CEA, NSE) were selected based on previous studies to establish the GEP model for the detection of SCLC. These biomarkers can be easily tested, even in developing regions, using two kits. For example, LDH and CRP, two important inflammation markers, are routinely tested in most hospitals in China, let alone electrolyte solution Na^+^, Cl^-^.

A previous study conducted by Flores, *et al*.[44,53,15] included 63 lung cancer patients, 87 non-cancer controls. The ANN model was trained with a set of biomarkers (Cyfra 21.1, CEA, CA125 and CRP) and achieved a correct classification rate of 88.9%, 93.3% and 90% in training, validation and testing phases, respectively. Feng, *et al*.[19] reached a prediction rate of 87.3% for the detection of lung cancers in a test phase using an ANN model with the above six biomarkers and 19 additional parameters, such as risk factors, symptoms, smoking, chemical exposure, kitchen environment, etc. Another study reached 90% specificity for the detection of lung cancer in the training set, based on a three-biomarker panel comprised of macrophage migration inhibitory factor (MIF), prolactin (PRL), and thrombospondin (THSP)[12]. According to the characteristic of “black-box” in ANN, we did not know how an ANN learns to perform its classification, merely giving a final results cause we fail to discern why it did not work[17]. Nevertheless, the GEP perform well even if there is large sophisticated data and offer a visual formula model. In our study, using the ROC curve to detect each sensitivity/specificity, we perceived that the area under the curve of Na^+^ and Cl^-^ is lower than others and the six biomarkers emerged the best. Then in GEP model 1, incorporating six biomarkers, successfully distinguished 281 of 300 tested samples with an accuracy of 93.75% (225/240) and 93.33% (56/60) for the training and test sets, respectively. Model 2, including four biomarkers, had slightly lower accuracies of 93.75% and 91.67% for the training and test sets, respectively. To confirm the excellent result in GEP, we repeated the models on ANN, Our results exhibited that the accuracy of GEP models performed higher than that in ANNs. The six biomarkers combined in MLP indicated a standout result that seem as to in GEP. Therefore, when compare the detection capability of ROC curve, GEP and ANN, GEP was proved to be the best algorithm which depend on model 1, otherwise, GEP model 2 with four biomarkers may be more suitable for screening SCLC in regions with extremely low incomes.

To confirm the GEP model 1 test, NSCLC patients have been included in the negative control with healthy subjects, the results were significantly worse than SCLC patients, also, there are significant differences in the concentrations of LDH, Na, Cl and NSE between SCLC and NSCLC. The results were enough to prove that the GEP model is suitable for the SCLC patients. Furthermore, The optimal GEP model 1 was used to make a comparison between early and late SCLC patients, the early SCLC model 1 acquired the accuracy of 92.37% (109/118) and 90% (27/30) for training and test set. It was close to total data accuracy. Meanwhile, the late SCLC model 1 performed well than early stage which represented the accuracy of 96.52% (112/116), 91.30% (27/29) for training and test set, respectively. The particular poor physical condition of the advanced patients may explain it. Generally, the accuracy of early stage was still good and GEP model 1 can be the optimal test for SCLC early detection.

In clinical examination, the serum CRP and LDH levels in SCLC patients are significantly higher than healthy people, but the serum sodium is much lower. The clinical significance of serum level of LDH has been proven to be a strong and independent predictive factor of median survival, both in limited and extensive disease stages of SCLC[54]. In addition, the correlation between inflammation and cancer risk has been reported in many studies. For example, tumor growth causes inflammation in tumor tissues, which can be regarded as an indicator of immune response to tumor antigens. In addition, cancer cells can increase the production of inflammatory cytokines, causing increased CRP levels in cancer patients[55,56]. CRP is a nonspecific acute-phase inflammatory response serum marker produced by hepatocytes and regulated by interleukin IL-6. The association between CRP and lung cancer has been widely investigated[57]. CEA is also an independent prognostic indicator associated with reduced survival in SCLC[34]. NSE has been regarded as the most sensitive tumor marker for SCLC at the time of diagnosis[35]. The close association of these biomarkers with SCLC is an important factor leading to the outstanding performance of our GEP models.

It has been reported that the syndrome of inappropriate antidiuretic hormone (SIADH) is the leading cause of hyponatremia and hypochloremia in hospitalized patients. Malignancy pulmonary diseases, particularly SCLC, usually lead to SIADH[31]. Therefore, clinical characteristics of hyponatremia and hypochloremia promote the search for underlying lung cancers by testing serum levels of biomarkers[58,59]. The other reasons for hyponatremia are the persistent natriuresis and inappropriately low aldosterone levels caused by increased levels of atrial natriuretic peptides (ANPs)[60], as well as involvement of the adrenal gland or the brain through metastases[61]. Thus, model l, which included Na^+^ and Cl^-^, as well as LDH, CRP, CEA, and NSE, had a slightly better performance for the detection of SCLC than model 2. GEP model 2 did not affect the detection accuracy by much, perhaps because paraneoplastic syndrome caused by SCLC is not common in clinical settings. However, GEP model 2 might be improved by adding more samples, new subjects, or keeping the normalizing criteria to train the model. In addition, clinical information, such as other biomarkers, nodules, and hemoptysis, etcetera, could also be included to improve the GEP performance.

In summary, we developed an effective GEP model incorporating six biomarkers to screen SCLC patients. This model and measurements of six biomarkers are convenient, economical, and can be widely used in less developed area. However, this model should be further tested and improved with more SCLC patients in different hospitals and regions. With the emergence of new predictive tumor markers, we are no longer going to select several determinate tumor markers joint detection, but going to obtain larger sample size, higher amount of information and larger scale on gene and protein levels. Moreover, due to the intricate parameter selection, the parameters in GEP algorithm may be optimized in the later research. Also, those serology tests couldn't replace lung biopsy to confirm a diagnosis, rather they serve as a good screening tool to auxiliary diagnosis. In addition, the economic cost of this GEP model needs to be comprehensively evaluated before it is widely applied in the clinical screening for SCLC.

## Supporting Information

S1 Dataset The six biomarkers data which were used in GEP models between SCLC patients and controls. (XLS)

S2 Dataset The six biomarkers data which were used in GEP models between NSCLC patients and SCLC patients. (XLSX)

S3 Dataset The six biomarkers serum concentrations of limited stage SCLC that used in GEP models. (XLSX)

S4 Dataset The six biomarkers serum concentrations of extensive stage SCLC that used in GEP models. (XLSX)
